# A Cyclic Undecamer Peptide Mimics a Turn in Folded Alzheimer Amyloid β and Elicits Antibodies against Oligomeric and Fibrillar Amyloid and Plaques

**DOI:** 10.1371/journal.pone.0019110

**Published:** 2011-04-19

**Authors:** Peter Hoogerhout, Willem Kamphuis, Humphrey F. Brugghe, Jacqueline A. Sluijs, Hans A. M. Timmermans, Janny Westdijk, Gijsbert Zomer, Claire J. P. Boog, Elly M. Hol, Germie P. J. M. van den Dobbelsteen

**Affiliations:** 1 Department of Vaccinology, Centre for Infectious Disease Control, National Institute for Public Health and the Environment (RIVM), Bilthoven, The Netherlands; 2 Netherlands Institute for Neuroscience (NIN), Astrocyte Biology & Neurodegeneration, Royal Netherlands Academy of Arts and Sciences, Amsterdam, The Netherlands; Leeds Institute of Molecular Medicine, United Kingdom

## Abstract

The 39- to 42-residue amyloid β (Aβ) peptide is deposited in extracellular fibrillar plaques in the brain of patients suffering from Alzheimer's Disease (AD). Vaccination with these peptides seems to be a promising approach to reduce the plaque load but results in a dominant antibody response directed against the N-terminus. Antibodies against the N-terminus will capture Aβ immediately after normal physiological processing of the amyloid precursor protein and therefore will also reduce the levels of non-misfolded Aβ, which might have a physiologically relevant function. Therefore, we have targeted an immune response on a conformational neo-epitope in misfolded amyloid that is formed in advance of Aβ-aggregation. A tetanus toxoid-conjugate of the 11-meric cyclic peptide Aβ(22–28)-YNGK′ elicited specific antibodies in Balb/c mice. These antibodies bound strongly to the homologous cyclic peptide-bovine serum albumin conjugate, but not to the homologous linear peptide-conjugate, as detected *in vitro* by enzyme-linked immunosorbent assay. The antibodies also bound—although more weakly—to Aβ(1–42) oligomers as well as fibrils in this assay. Finally, the antibodies recognized Aβ deposits in AD mouse and human brain tissue as established by immunohistological staining. We propose that the cyclic peptide conjugate might provide a lead towards a vaccine that could be administered before the onset of AD symptoms. Further investigation of this hypothesis requires immunization of transgenic AD model mice.

## Introduction

Alzheimer's disease (AD) is a neurodegenerative disorder and the most common cause of dementia in elderly [1], [2]. A characteristic of the disease is formation of plaques in the brain or in brain blood vessels. These plaques originate from a membrane-bound protein, amyloid precursor protein (APP). An α-helical fragment of 39–42 amino acid residues is cleaved by β- and γ-secretases from APP thus forming a soluble amyloid β (Aβ) peptide. Soluble Aβ initially adopts an extended conformation but at high concentrations, soluble Aβ will undergo conformational changes and form oligomers, protofibrils, and fibrils. In AD, fibrillar Aβ is deposited in the brain as amyloid plaques, which is one of the main neuropathological hallmarks of the disease. However, accumulating studies suggest that the soluble oligomeric Aβ instead of insoluble Aβ in amyloid plaques is the culprit in AD [3]–[8] and therapeutic approaches aimed at preventing the formation of these oligomeric isoforms may be able to reduce the progression of the disease. In line with this concept, immunization of transgenic mice [9] with a suspension of “pre-aggregated” Aβ(1–42) and the adjuvant quillaja saponin 21 appeared to be beneficial. Based on these results, a phase I clinical trial was started. Antibodies present in human sera recognized plaques and Aβ deposits in brain blood vessels [10]. The antibodies did not recognize APP or soluble Aβ. In the following phase II clinical trial, 20% of the vaccine recipients generated anti-Aβ antibody titers. Unfortunately, this trial had to be terminated since 6% of the patients developed meningoencephalitis as a vaccine-related side effect. This side effect was caused by a cellular inflammatory reaction, attributed to a T helper cell type 1 response to epitopes located in the central and C-terminal part of Aβ(1–42) [11], [12].

Multiple ongoing studies aim at improving the Aβ vaccination strategy [13]–[18]. The use of T helper cell type 2 directing adjuvants [19], [20] or the use of formulations without any adjuvant [21] are under investigation. In addition, it has been proposed to use C-terminally truncated Aβ peptides [22], [23] or peptide mimics (affitopes) of the N-terminus [24]. Antibodies induced by Aβ(1–42) are dominantly directed against the linear N-terminal epitope [25], [26], although generation of conformation-specific antibodies against other regions within aggregated Aβ has been reported [27]. A disadvantage of a vaccine against the N-terminus of Aβ is that it will interfere with the normal physiological processing of APP. It may not be without risk to administer such a vaccine before onset of symptoms of AD.

**Figure 1:**
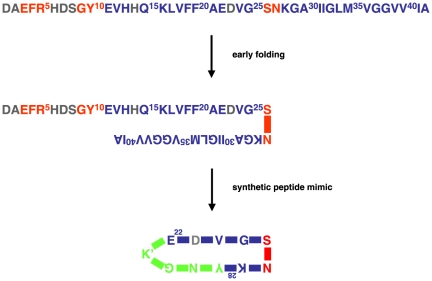
Early folding of human amyloid β (Aβ), around S26 and N27. The figure is a simplification of the model designed by Olofsson *et al.* [28]. Residues in red are solvent accessible; residues in blue (and to a lesser extent in gray) are shielded from the solvent. The peptide cyclo[Aβ(22–28)-YNGK′] is a mimic for misfolded Aβ. YNGK′ is a turn-stabilizing sequence and K′ is a side-chain-modified lysyl residue for selective conjugation to a protein carrier. Other YNGK′-containing cyclic peptides prepared (23–28, 24–29, 25–30, 21–27, 22–28, 23–29, 24–30, and 25–31) did not mimic misfolded Aβ.

By targeting an immune response exclusively on misfolded Aβ the undesired response against the N-terminus of Aβ may be avoided all together. A structural model of fibrillar Aβ(1–42) predicts folding of monomeric Aβ(1–42) into a cross-β unit. Two antiparallel extended β-strands, residues 11–25 and 28–42, are connected via a sharp bend around amino acid residues S26 and N27 [28]. Fig. 1 shows a simplification of the original model. A recent study of a particular oligomer of N-Met-Aβ(1–42) suggests a bend around the sequence V^24^-G-S-N^27^ [29].

The models of folded Aβ show some resemblance with the β-turn structure in surface loops of meningococcal outer membrane protein PorA. Previously, we have stabilized the β-turn conformation of small meningococcal peptides by adding an artificial sequence YNGK′, in which K′ is a modified lysine residue for selective conjugation to a carrier protein, followed by main chain (“head to tail”) amide cyclization [30], [31]. Likewise, it appeared possible to prepare small cyclic peptides mimicking the turn in folded Aβ. A panel of cyclic decameric and undecameric peptides spanning six or seven residues from the region 21–31 of Aβ and YNGK′ was prepared and conjugated to tetanus toxoid (TTd). The conjugates were used for immunization of mice. A tetanus toxoid-conjugate of one of the peptides, cyclo[Aβ(22–28)-YNGK′], elicited antibodies that specifically recognized misfolded Aβ.

## Results and Discussion

### Antigen preparation

Oligomeric (≈35 kD) and fibrillar Aβ(1–42) were prepared according to published procedures [32]. A well-established method [33] was used for synthesis of a panel of cyclic decameric and undecameric peptides spanning six or seven residues from the region 21–31 (AEDVG^25^SNKGA^30^I) of Aβ (*i.e.* 23–28, 24–29, 25–30, 21–27, 22–28, 23–29, 24–30, and 25–31) and the artificial stabilizing sequence YNGK′. Linear Ac-K′-Aβ(21–31)-NH_2_ was prepared to serve as control. The peptides obtained were conjugated to tetanus toxoid (TTd) to give experimental vaccines or to bovine serum albumin (BSA) to give coating antigens for enzyme-linked immunosorbent assay (ELISA ) [34].

### Immunization of mice

Balb/c mice were immunized twice with oligomeric or fibrillar Aβ(1–42) without adjuvant or with the different amyloid peptide/TTd-conjugates using aluminum phosphate as adjuvant. The pooled sera from each group immunized with peptide conjugate were analyzed by ELISA using plain TTd, homologous peptide/BSA-conjugate and oligomeric or fibrillar Aβ(1–42) as coating antigens. The sera showed a good antibody titer against TTd and the homologous cyclopeptide (titers of 10^4^–10^5^ were obtained, data not shown).

**Figure 2:**
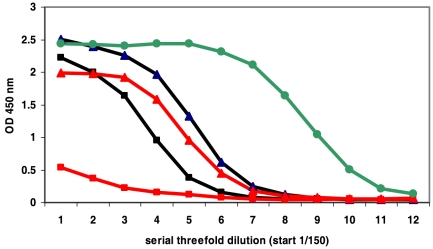
Binding of anti-cyclopeptide antibodies to oligomeric amyloid β (Aβ) as determined by ELISA. Threefold serial dilutions (starting from 1/150) of pooled antisera obtained were tested. Antisera were raised by immunization with oligomeric (black ▴) or fibrillar (black ▪) Aβ(1–42) and TTd-conjugates of cyclo[Aβ(22–28)-YNGK′] (red ▴) or linear K′-Aβ(22–28)-YNG (red ▪). Monoclonal antibody 6E10 (green •) served as control. Antibody binding to fibrillar Aβ(1–42) gave comparable results.

**Figure 3:**
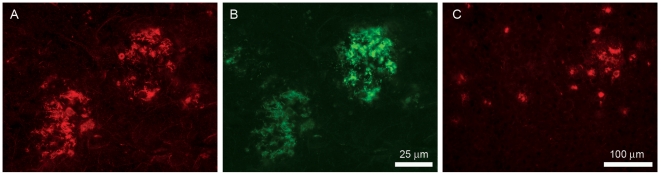
Immunohistochemical staining of brain tissue. **A**, a brain section (hippocampus) of an 85-year-old female AD patient (NBB 1999-030) was stained with mouse anti-cyclo[Aβ(22–28)-YNGK′] serum and (**B**) co-stained with an antibody against APP (6E10). The neuropathological diagnosis of the is patient is Braak stage for neurofibrillary tangles 6 and for amyloid B [43]. **C**, immunostaining of a plaque-loaded cortex of a 9 month old APPswe/PS1dE9 transgenic mouse.

**Figure 4:**
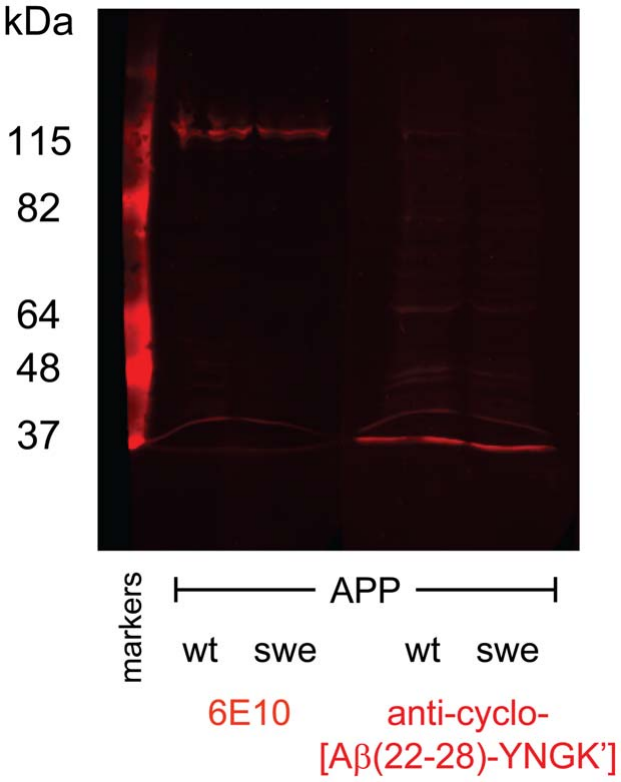
Western blot of a lysate of SW13 adrenal cells transfected with amyloid precursor protein (APP). The cells were transfected with either human APP^695^wt (wild type) or with APP^695^swe (Swedish mutation). Monoclonal antibody 6E10 is specific for the largely unstructured N-terminus of amyloid β (Aβ) and recognized both APPs (amino acid residues 2–8 in Aβ correspond to residues 598–604 in APP^695^). Anti-cyclo[Aβ (22–28)-YNGK′] antibodies stained neither of the APPs under the denaturing conditions of the assay and thus seem to recognize a conformational epitope in Aβ.

Six out of eight pooled anti-cyclo-Aβ peptide antisera were not cross-reactive with oligomeric or fibrillar Aβ(1–42) in ELISA or a dot blot assay. Antibodies against cyclo[Aβ(23–29)-YNGK′] showed a weak cross-reactivity. However, anti-cyclo[Aβ(22–28)-YNGK′] antibodies were strongly cross-reactive with oligomeric or fibrillar Aβ(1–42) in ELISA (Fig. 2) and the antibodies recognized Aβ deposits in AD mouse and human brain tissue (Fig. 3). The anti-peptide antibodies did not recognize full length APP in Western blot assay (Fig. 4).

The cyclization of the peptide was essential to produce a functional antigen. Conjugates of linear control peptides, *i.e.* Ac-K′-Aβ(21–31)- NH_2_ or the fully homologous Ac-K′-Aβ(22–28)-YNG-NH_2_, induced good anti-peptide antibody titers but the antibodies failed to recognize oligomeric or fibrillar Aβ(1–42) (Fig. 2). The incorporation of the artificial sequence YNGK′ in the cyclopeptide was essential to mimic the turn in folded Aβ(1–42). Incorporation of the sequence GAIK′, *i.e.* Aβ(29–31)-K′, instead of YNGK′ did not provide a turn-mimicking peptide, since antibodies against cyclo[Aβ(22–31)-K′] were not functional.

The anti-cyclo[Aβ(22–28)-YNGK′] antibodies were very specific for the common Aβ sequence. Amyloid β with the Dutch, Arctic, or Italian mutations (E22Q, E22G, or E22K, respectively) was not recognized. Unrelated but partially homologous proteins like the islet amyloid polypeptide (amylin)or the human Wiskott-Aldrich Syndrome protein (WASP) were also not recognized (data not shown).

Finally, antibodies elicited with oligomeric or fibrillar Aβ(1–42) were non-reactive with cyclo[Aβ(22–28)-YNGK′]-BSA. This was expected since antibodies induced by Aβ(1–42) are directed against the N-terminus [25], [26].

**Figure 5:**
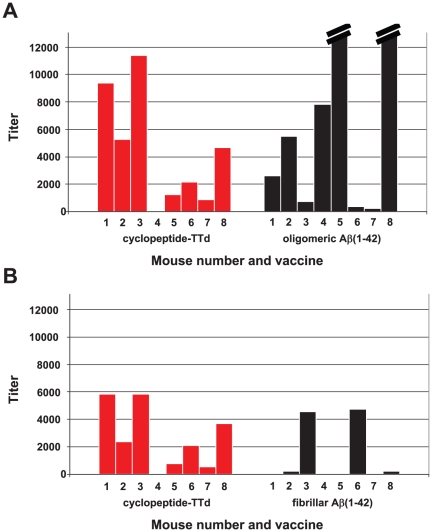
IgG response of individual Balb/c mice against Aβ (1–42) as determined by ELISA. As indicated on the x-axis, groups of eight mice were immunized with cyclo[Aβ (22–28)-YNGK′]-TTd, oligomeric Aβ(1–42), or fibrillar Aβ (1–42). **A**, coating antigen: oligomeric Aβ (1–42); **B**, coating antigen: fibrillar Aβ (1–42). A mouse with a serum titer ≤100 is considered to be a non-responder. The two titers out of range in the right panel of **A** have values of 40**×**10^3^ and 50**×**10^3^. The figure demonstrates that anti- cyclo[Aβ (22–28)-YNGK′] antibodies recognize oligomeric and fibrillar Aβ (1–42).

The cross-reactivity of anti-cyclo[Aβ(22–28)-YNGK′] antibodies with oligomeric Aβ(1–42) was confirmed after a second immunization experiment. Individual sera of mice immunized with cyclo[Aβ(22–28)-YNGK′]-TTd were analyzed this time. All sera (8/8) gave a good response against TTd and the homologous peptide (titers 10^4^–10^5^, data not shown), but not all individual sera were clearly cross-reactive with oligomeric or fibrillar Aβ(1–42). Five sera showed titers ≥2000 (Fig. 5), whereas one serum showed no response (titer<100). Two immunizations with oligomeric Aβ(1–42) gave two sera with titers as high as 40**×**10^3^ and 50**×**10^3^ on the homologous coating. Three titers were considered to be intermediate (2000–8000) and three titers were low (100–2000).

Two immunizations with fibrillar Aβ(1–42) gave only two sera with titers **≥**2000 on the homologous coating, two very low responders and four non-responders (titer<100). Poor responsiveness to aggregated Aβ(1–42) was found earlier in clinical and animal studies [35], [36].

In conclusion, cyclo[Aβ(22–28)-YNGK′] mimics a conformational epitope in folded Aβ(1–42) that normally does not induce an antibody response. Cyclo[Aβ(22–28)-YNGK′]-conjugate may be an interesting vaccine candidate against AD. Since the response is directed specifically to misfolded Aβ, it is unlikely that vaccination will interfere with normal physiological processing of the amyloid precursor protein. Thus, vaccination might be possible at an early stage of AD symptoms or before. This seems to be indicated since plaque deposits have already reached a near maximum level at a mild stage of AD [37].

Further studies will aim at improvement of the immune response to cyclo[Aβ(22–28)-YNGK′]-TTd by variation of the dose of cyclopeptide-conjugate, the adjuvant, and the number of administrations, preferably in transgenic AD model mice.

## Materials and Methods

### Ethics statement

Tissue from AD patients was obtained from the Netherlands Brain Bank (NBB; Amsterdam, The Netherlands). The NBB performs brain autopsies with short post-mortem intervals, and the brain donors have given written informed consent for using the tissue and for accessing the extensive neuropathological and clinical information for scientific research, in compliance with ethical and legal guidelines [38]. The study was approved by the independent Review Board (“Medisch Ethische Toetsingscommissie, METc”) of the VU University Medical Center, Amsterdam, The Netherlands.

Morphological studies of mouse brain tissue were approved by the Ethical Committee on Animal Experiments of the Royal Netherlands Academy of Arts and Sciences (approval ID: NIN 06.51).

Immunization of mice was approved by the Ethical Committee on Animal Experiments of the National Institute of Public Health and the Environment (approval ID: 2007 00331 and 2008 00179), as required under Dutch Law on the use of laboratory animals. All efforts were made to minimize suffering of the animals.

### Materials

Human Aβ(1–42) (Fig. 1), hAβ(1–40/42) analogues, and hIAPP were purchased from AnaSpec (Freemont, CA, USA). AlPO_4_ suspension (1.5 mg/ml), pH 7.0, and tetanus toxoid (TTd) were produced by the RIVM. Monoclonal antibody 6E10 against Aβ(1–17), was purchased from Abcam (Cambridge, MA, USA) or Covance Research Products, Dedham, MA, USA).

### Disaggregation of Aβ(1–42)

Lyophilized Aβ(1–42) was dissolved in trifluoroacetic acid at a concentration of 1.0 mM, left to stand at room temperature for 1 h and dried under a stream of nitrogen and, thereafter, in a vacuum (1 mm Hg) for 15 min. The peptide was then redissolved in hexafluoroisopropanol at a concentration of 1.0 mM and, after 1 h of incubation at room temperature, dried as described above [39]. The peptide was stored at −20°C for 18–20 h.

### Preparation of oligomeric or fibrillar Aβ(1–42)

Disaggregated of Aβ(1–42) was dissolved in dimethyl sulfoxide at a concentration of 5.0 mM, diluted 50-fold with either phosphate buffered saline (PBS), pH 7.2, or 10 mM hydrochloric acid. The solution in PBS was incubated at 4°C for 24 h (to give oligomers), whereas the solution in 10 mM HCl was incubated at 37°C for 24 h (to give fibrils) [32].

### Peptide synthesis, purification, and conjugation

The α-(2,4-dimethoxybenzyl) ester of N^α^-fluorenylmethoxycarbonyl-L-aspartic acid (Fmoc-Asp-ODmb) was coupled through its side-chain to a polymer for the synthesis of peptide amides (for later conversion of the starting Asp into Asn). Synthesis of cyclic peptides, like cyclo[GK′EDVGSNKYN]≡cyclo[EDVGSNKYNGK′] in which K′ is *N* ^ε^-(*S*-acetylmercaptoacetyl)lysyl, was then performed as described earlier [33]. The following Aβ-derived and YNGK′-extended cyclopeptides were prepared: 23–28, 24–29, 25–30, 21–27, 22–28, 23–29, 24–30, and 25–31. Linear Ac-K′-Aβ(22–31)-NH_2_ and linear Ac-K′-Aβ(22–28)-YNG-NH_2_ were also prepared. The peptides were purified by reverse-phase high performance liquid chromatography and coupled to either bromoacetylated tetanus toxoid or maleimidyl-modified bovine serum albumin (BSA) (modifying reagent: NHS-PEO_2_-Maleimide, Pierce) [34].

### Immunization of mice

Groups of eight female Balb/c mice of 6–8 weeks of age were immunized with 25 µg oligomeric or fibrillar Aβ(1–42) in 0.3 ml phosphate buffered saline (PBS), pH 7.2, without adjuvant or with 50 µg of peptide/TTd-conjugate and 75 µg of AlPO_4_ in 0.3 ml PBS on days 0 and 28. The mice were bled on day 42 under oxygen/nitrous oxide/isoflurane anesthesia.

### Western blot

Human carcinoma SW13 cells [40] were transfected with APP^695^wt (wild type) and APP^695^swe (Swedish mutation) and proteins were isolated by homogenization in 0.1 M NaCl, 0.01 M Tris HCl, 0.001 M EDTA, pH 7.6, with proteinase inhibitors. The samples were supplemented with 2× loading buffer (100 mM Tris pH 6.8, 4% SDS, 20% glycerol, 0.2 M DTT, 0.006% bromophenol blue) and run on a 7.5% SDS-PAGE gel. The gel was semi-dry blotted on nitrocellulose and probed overnight with the 6E10 antibody (1∶1000) or the mouse anti-cyclo[Aβ(22–28)-YNGK′] serum (1∶500). After washing, the blots were incubated with anti-mouse Cy5 (1∶300), bands were visualized with the Odyssey Infrared Imaging System.

### Dot blot

Dilutions of cell lysates (WASP**^pos^** HL60 cells and WASP**^neg^** Vero cells) in PBS were spotted (1 µl/spot) on polyvinylidene difluoride membrane (Immobilon-P, pore size 0.45 µM, Millipore). The blots were incubated with mouse anti-cyclo[Aβ(22–28)-YNGK′] serum, control monoclonal antibody 6E10, or polyclonal anti-WASP antibodies. After washing, the blots were incubated with horseradish peroxidase-conjugated 2^nd^ antibody, and visualized with GZ11 signal reagent [41].

### ELISA

Microtiter plates (Greiner 655092) were coated with Aβ(1–42) or peptide-BSA conjugates. Freshly prepared Aβ(1–42) oligomers or fibrils were diluted to a final concentration of 2.5 µM (11.3 µg/ml) in 0.04 M sodium carbonate/bicarbonate buffer, pH 9.7. Peptide-BSA conjugates in phosphate buffered saline, pH 7.2 (PBS), had a total protein concentration of 0.5 µg/ml. Aliquots (100 µl) of these solutions were transferred into wells of the plates. The plates were incubated for 90 min at 37°C. The plates were further processed as described earlier [42]. Titers were calculated as the reciprocal serum dilution at 50% of the maximum optical density (OD_50_).

### Immunohistochemical staining

Human brain sections of the hippocampus of several donors with Alzheimer disease, Braak 5 or 6 [43], were used (Netherlands Brain Bank). In addition, brain sections of 9 month old APPswe/PS1dE9 [42] mice with a significant plaque deposition were used. Cryosections (10 µm) were cut from unfixed, directly frozen tissue, thaw-mounted, dried for 1 hour and stored in a sealed box at −20 C. For immunostaining, sections were fixed in 4% PFA-PBS solution for 10 min, washed in 0.05 M phosphate buffer (PB) for 10 min with 2 exchanges and blocked with 10% normal donkey serum (NDS) +0.4% Triton X-100 in 0.05 M PB for 1 hour at RT. The blocking solution was discarded and diluted mouse sera (1∶300; first antibody) in 3% NDS +0.4% Triton X-100 in 0.05 M PB was added and incubated O/N at RT. Sections were washed with 0.05 M PB; at least 30 min with one or more exchanges. Then sections were incubated with Donkey-anti-Mouse ∼Cy3 1∶1400 in 0.05 M PB for 2 hours. Sections were washed with 0.05 M PB; at least 30 min with one or more exchanges. Sections were sealed in Vectashield with Dapi (Vector). Mouse Monoclonal 6E10 to beta amyloid 3–8 (Signet-Covance) was used as positive control (1∶15,000).
